# Loneliness, Circulating Endocannabinoid Concentrations, and Grief Trajectories in Bereaved Older Adults: A Longitudinal Study

**DOI:** 10.3389/fpsyt.2021.783187

**Published:** 2021-12-08

**Authors:** Minhi Kang, Luisa Bohorquez-Montoya, Timothy McAuliffe, Stacy A. Claesges, Nutta-On Blair, Garrett Sauber, Charles F. Reynolds, Cecilia J. Hillard, Joseph S. Goveas

**Affiliations:** ^1^Department of Psychiatry and Behavioral Medicine, Medical College of Wisconsin, Milwaukee, WI, United States; ^2^Department of Pharmacology and Toxicology, Medical College of Wisconsin, Milwaukee, WI, United States; ^3^Neuroscience Research Center, Medical College of Wisconsin, Milwaukee, WI, United States; ^4^Department of Psychiatry, University of Pittsburgh School of Medicine, Pittsburgh, PA, United States; ^5^Institute for Health and Equity, Medical College of Wisconsin, Milwaukee, WI, United States

**Keywords:** loneliness, grief, bereavement, prolonged grief disorder (PGD), older adult, *N*-arachidonoylethanolamine, 2-arachidonoylglycerol, endocannabinoid

## Abstract

**Background:** Loneliness is one of the most distressing grief symptoms and is associated with adverse mental health in bereaved older adults. The endocannabinoid signaling (ECS) system is stress-responsive and circulating endocannabinoid (eCB) concentrations are elevated following bereavement. This study examined the association between loneliness and circulating eCB concentrations in grieving older adults and explored the role of eCBs on the association between baseline loneliness and grief symptom trajectories.

**Methods:** A total of 64 adults [grief with high loneliness: *n* = 18; grief with low loneliness: *n* = 26; and healthy comparison (HC): *n* = 20] completed baseline clinical assessments for the UCLA loneliness scale. In grief participants, longitudinal clinical assessments, including the Inventory of Complicated Grief and 17-item Hamilton Depression Rating scales, were collected over 6 months. Baseline circulating eCB [*N*-arachidonoylethanolamine (AEA) and 2-arachidonoylglycerol (2-AG)] concentrations were quantified in the serum using isotope dilution, liquid chromatography-mass spectrometry; cortisol concentrations were measured in the same samples using radioimmunoassay.

**Results:** Circulating AEA concentrations were higher in severely lonely grieving elders than in HC group; cortisol concentrations were not different among the groups. Cross-sectionally, loneliness scores were positively associated with AEA concentrations in grievers; this finding was not significant after accounting for depressive symptom severity. Grieving individuals who endorsed high loneliness and had higher 2-AG concentrations at baseline showed faster grief symptom resolution.

**Conclusions:** These novel findings suggest that in lonely, bereaved elders, increased circulating eCBs, a reflection of an efficient ECS system, are associated with better adaptation to bereavement. Circulating eCBs as potential moderators and mediators of the loneliness-grief trajectory associations should be investigated.

## Introduction

Loss of a loved one is one of the most traumatic and painful events an individual will experience during his/her lifetime. Over 2.5 million people die annually in the United States. Using an estimated average of nine bereaved persons for each death, ~22.5 million people will experience acute grief each year (1). Bereavement is particularly experienced at a greater frequency by older adults and most bereaved older individuals adapt to the loss and return to their pre-loss functioning within 1 year. However, in about 10% of bereaved individuals, grief follows an intense, protracted, and disabling trajectory resulting in prolonged grief disorder (PGD), a clinical condition that is diagnosed 12-months post-loss (2, 3). PGD is associated with declines in physical health and cognition, and an elevated risk of adverse health behaviors (e.g., increased alcohol and tobacco use), hypertension, cardiac events, cancer, hospitalizations, and premature mortality from different causes, including suicide (2, 3). The consequences of PGD are therefore enormous; however, little is known regarding modifiable factors and related biological mechanisms that are associated with the clinical course in bereaved older individuals.

Loneliness is a modifiable factor that is commonly reported by older individuals during the first year after bereavement. About 70% of older widow(er)s describe loneliness as the single most difficult challenge to cope with daily (4). Loneliness is a major social determinant that increases the risk of adverse mental health outcomes in older adults (5), including after bereavement (6). Intense loneliness is also one of the main associated symptoms of PGD (7). Despite the negative impact of loneliness in bereaved elders, the biological processes through which loneliness during the first-year post-loss is associated with grief trajectories and the development of PGD are unknown.

The endocannabinoid signaling (ECS) system is a neuromodulatory system consisting of cannabinoid receptors (CB1 and CB2), their lipid ligands, the endocannabinoids (eCBs) *N*-arachidonoylethanolamine (AEA or anandamide) and 2-arachidonoylglycerol (2-AG), and the synthetic and degradative enzymes for the eCBs (8, 9). The ECS system is responsive to acute traumatic stress and modulates the effects of stress on the brain (10, 11). The eCBs are produced on demand; it is hypothesized that by modulating the synaptic activity in the emotion regulation and reward processing brain circuits (11–13), eCBs regulate behavioral responses and reduce the incidence of trauma- and stressor-related psychopathologies (8). We thus posit that the ECS system is a candidate biological mechanism of PGD because this clinically diagnosable condition arises following bereavement, an acute traumatic, and stressful event.

The eCBs, AEA, and 2-AG, can be reliably measured in the human serum and plasma. It is hypothesized that the brain is a source of circulating eCBs, although other organs and tissues can also synthesize and release the eCBs into circulation (14). Consistent with the ECS hypothesis, acute psychological and physical stress-induced increases in circulating eCBs are reported in healthy humans (15–19). Elevated circulating AEA concentrations are also found in grieving older individuals who are within 1 year of bereavement (20). On the contrary, circulating eCB concentrations are reduced in healthy individuals who are exposed to chronic stress (21) as well as in those with post-traumatic stress disorder (22, 23). In the only previous study of the effects of social disconnection on circulating eCBs, 2-AG concentrations were found to be significantly reduced in healthy individuals after exposure to 520 days of prolonged isolation and confinement (21). These findings are consistent with the preclinical evidence of a hypoactive ECS system in response to chronic stress (9, 24), and a blunted 2-AG response in PTSD patients following stress exposure (18, 25).

In this pilot study, we aimed to *cross-sectionally* examine (1) circulating eCB concentrations in bereaved participants with varying intensity of loneliness and non-bereaved healthy comparison (HC) participants, and (2) the association between loneliness and circulating eCB concentrations in grieving individuals. We further examined *longitudinally* the role of circulating eCB concentrations on the association between baseline loneliness and grief symptom trajectories over 26 weeks (i.e., 6 months). Given the results of acute grief and PGD studies (26, 27), and evidence of a close association between the ECS and hypothalamic-pituitary-adrenal (HPA) axis (8), we also measured cortisol concentrations and examined the relationships between cortisol and AEA and 2-AG concentrations.

## Methods

### Study Participants

A total of 75 adults, aged 50 years and older (age range: 51–89 years), completed baseline (week 0) clinical assessments, including the 20-item UCLA loneliness scale-version 3 (UCLA-3) (28), and had a fasting blood draw. Participants were enrolled into (1) grief (*n* = 54) if they were within 13 months following the death of a loved one, or (2) HC (*n* = 21) groups. These participants were recruited through advertisements, and referrals from grief groups and hospice counselors.

*For this study*, all grief participants who consented to a 6-month longitudinal study (*n* = 44) were included in the analysis (see inclusion/exclusion criteria). Of the 21 participants who were classified as HC, one participant endorsed high loneliness and was not included; the remaining individuals endorsed low to moderate feelings of loneliness and comprised the final HC sample (*n* = 20) (see inclusion/exclusion criteria). All participants provided written informed consent according to the Institutional Review Board-approved protocols.

### Assessment Procedures

#### Baseline (Week 0) Visit

All grief (*n* = 44) and HC (*n* = 20) participants completed baseline clinical assessments, including a Structured Clinical Interview for DSM-5 Research Version (29). Sociodemographic characteristics, medical and psychiatric histories, and medication history were obtained, and a neurological examination was performed.

Perceived loneliness, the independent variable of interest, was measured using the UCLA-3 (28). A battery of other tests was completed by all participants, including the 17-item Hamilton Depression Rating Scale (HAM-D) (30), Hamilton Anxiety Scale (HAM-A) (31), the Cumulative Illness Rating Scale-Geriatric version (CIRS-G) (32), modified Hachinski Ischemic Scale (HIS), the Scale of Suicidal Ideation (SSI) (33), Mini-Mental State Exam (MMSE) (34), and Mattis Dementia Rating Scale-2 (35). Psychotropic medication history was documented. The grief participants also completed the 19-item Inventory of Complicated Grief (ICG) scale (36), and the relationship to the deceased and the time since the loss (TSL) were collected.

A one-time fasting blood draw was obtained within 20.5 ± 13 days of the baseline clinical visit (Grief: 20.5 ± 15 days; HC: 21 ± 9.5 days).

#### Longitudinal Visits

Grief participants (*n* = 44) completed follow-up clinical assessments, including the ICG and HAM-D scales, at weeks 8, 16, and 26 (i.e., 6 months), respectively.

### Inclusion and Exclusion Criteria

All participants had to score <4 on the modified HIS, and a score >24 on the MMSE. Exclusion criteria included a lifetime history of bipolar or psychotic disorders; alcohol or substance use disorders during the past 5 years; acute suicidality (assessed using the SSI and the third HAM-D item score or judged by a clinician); a history of neurological illnesses, including seizures, stroke, dementia of any etiology, severe head injury, brain tumor, etc.; and delirium/unstable medical conditions determined using the CIRS-G score of 4 in any category.

#### Grief Participants

Participants were included in the grief group if they were within 13 months following the death of a loved one and consented to participate in the longitudinal study (*n* = 44). Grief participants who met current DSM-5 criteria for depressive, anxiety, or post-traumatic stress disorders were not excluded if the onset of psychopathology followed bereavement. Antidepressant medications and low doses of benzodiazepines (i.e., diazepam 5 mg equivalent daily dose of either lorazepam or clonazepam) were allowed.

Based on prior literature (37, 38), grief participants were further classified into two groups using a UCLA-3 score cutoff of 43: those with an UCLA-3 score ≤43 (*n* = 26) were considered to have low to moderate loneliness (Grief-LL) and those with an UCLA-3 score >43 (*n* = 18) were considered to have severe or high loneliness (Grief-HL).

#### HC Participants

Inclusion criteria for HC participants (*n* = 20) included those with no lifetime history of any psychiatric illnesses, no history of the death of a loved one within 13 months of clinical visit, no current psychotropic medication use, and a UCLA-3 score ≤43.

### Experimental Procedures

#### Circulating eCB and Cortisol Concentrations

A one-time fasting blood draw at baseline was conducted in all participants between 7:00 and 11:00 a.m. Blood was refrigerated after collection and serum was separated by centrifugation within 60 min and stored in 3 ml aliquots at −80°C. Serum concentrations of AEA and 2-AG were determined in extracted lipids from serum samples using stable isotope-dilution, liquid chromatography-mass spectrometry quantification methods as described previously (18, 20). Cortisol concentrations (μg/dl) were measured in triplicate using 25 μl of the same samples using a radioimmunoassay kit from MP Biomedical (0722110-CF) following manufacturer's instructions. Sample and standard disintegrations per min were converted to percent cortisol bound and serum cortisol concentrations were determined *via* interpolation from a standard curve of log concentration vs. percent bound.

#### Clinical Outcome Measure

ICG is a 19-item, self-report questionnaire that has good to excellent psychometric properties and assesses symptoms of PGD. ICG has been previously utilized in PGD treatment studies, and the total scores range from 0 to 76; a score of 30 or higher is indicative of PGD (36).

### Statistical Analysis

Demographics and clinical characteristics were compared between the HC, Grief-LL, and Grief-HL groups using the Kruskal-Wallis *H*-test. Chi-square tests were used to examine gender and race differences.

ANCOVA was used to compare circulating eCB and cortisol concentrations between HC, Grief-LL, and Grief-HL groups, after adjusting for age and gender. Specifically, two weighted effect contrasts, one comparing between the Grief-HL and Grief-LL groups, and the second comparing between the Grief-HL and HC groups, were tested within the general linear model framework using robust standard error estimation. The weighted contrasts are used to adjust for unequal sample sizes for each contrast considering modest sample sizes in the groups.

Linear regression models examined the associations between loneliness and circulating eCB concentrations in grief participants using three separate models: Model 1–adjusting for age, gender, and TSL; Model 2–adjusting for covariates in model 1 plus ICG; and Model 3–adjusting for covariates in model 2 plus HAM-D. General linear model was used to explore the main and interaction effects of ICG and loneliness, and HAM-D and loneliness, on serum AEA and 2-AG concentrations. Residual analysis was conducted to ensure that the linear model assumptions were satisfied. Additionally, linear regression models explored the cross-sectional associations between mean cortisol concentrations (μg/dl) and (1) AEA, and (2) 2-AG concentrations without any covariate adjustment.

For the longitudinal analyses, mixed-effects linear models examined the association of a three-way multiplicative interaction of loneliness, circulating eCB concentration (i.e., AEA or 2-AG), and time/visit with longitudinal ICG scores over 26 weeks in grief participants, controlling for the main terms (loneliness, circulating eCB, time/visit) and their interactions with time/visit. Age, gender, and TSL were included in both models. For these analyses, loneliness scores were dichotomized to high (score > 43) or low (score ≤ 43). Circulating eCB concentrations were dichotomized based on the sample median into (1) AEA concentrations: high (>2.0 pmol/ml) or low (≤2.0 pmol/ml); and (2) 2-AG concentrations: high (>18.7 pmol/ml) or low (≤18.7 pmol/ml). Generalized estimating equations were used to compare changes in ICG over 26 weeks in high and low loneliness and high and low serum 2-AG concentrations groups.

The overall significance was set at *p* < 0.05 (two-tailed). All analyses were conducted using the IBM SPSS statistics Version 26.0 (IBM, Armonk, NY, USA).

## Results

### Demographic and Clinical Characteristics

Baseline demographic and clinical characteristics are shown in Table 1. Compared to HC and Grief-LL, those grievers with high loneliness were younger (HC > Grief-HL, *p* = 0.003; Grief-LL > Grief-HL, *p* < 0.001). The groups did not differ in other demographic characteristics or cognitive and CIRS-G measures. Grief and HC groups differed in UCLA-3, HAM-D, and HAM-A scores. Compared to Grief-LL, those with high loneliness had higher ICG scores (*p* = 0.004); grief groups did not differ in TSL or relationship to the deceased. Among the grief participants, 14 (78%) with high loneliness and 7 (27%) with low loneliness met the SCID major depressive disorder (MDD) criteria. About 70% of grief participants either lost a spouse, significant other or child. There was no significant difference in the relationship with the deceased between high and low loneliness grief groups. Sixteen grief participants were taking antidepressants [Grief-HL group: *n* = 9, with a combination (*n* = 5) therapy and monotherapy (n = 4); Grief-LL group: *n* = 7, with combination therapy (*n* = 2) and monotherapy (*n* = 5)].

**Table 1:** Baseline demographic and clinical characteristics.

**Variable**	**Grief**		**Healthy comparison (*n* = 20)**	**df**	**Statistic**	***p*-value**
	**High loneliness (*n* = 18)**	**Low loneliness (*n* = 26)**				
Demographics						
Age (years)	60.5 ± 7.5	70.5 ± 9.7	70.6 ± 9.0	2	H = 13.1	**0.001** ^a^
Sex (F/M)	12/6	18/8	19/1	NA	FET = 6.0	0.06
Race (*n*)				NA	FET = 4.9	0.13
White	16	25	20			
Black	2	0	0			
Am Indian	0	1	0			
Years of education, mean (SD)	15.4 ± 3.3	15.9 ± 3.0	16.6 ± 2.5	2	H = 1.8	0.42
Grief measures						
ICG, mean (SD)	36.4 ± 13.3	23.7 ± 12.2	NA	1	H = 8.2	**0.004**
Time since loss (days), mean (SD)	188.1 ± 106.9	139.7 ± 83.5	NA	1	H = 2.0	0.16
Relationship with the deceased			NA	NA	FET = 6.9	0.06
Spouse/significant other	6 (33%)	16 (62%)	–		–	–
Daughter?/son	4 (22%)	5 (19%)	–		–	–
Mother?/father	7 (39%)	2 (8%)	–		–	–
Other	1 (6%)	3 (12%)	–		–	–
Cognitive/medical/psychiatric measures						
MMSE, mean (SD)	28.2 ± 1.8	28.6 ± 1.4	28.9 ± 1.3	2	H = 1.4	0.50
DRS-2 total, mean (SD)	139.3 ± 3.7	141.1 ± 2.8	141.2 ± 1.6	2	H = 3.9	0.14
CIRS-G, mean (SD)	6.3 ± 3.7	5.2 ± 3.2	4.9 ± 2.9	2	H = 2.1	0.36
HAM-A, mean (SD)	13.1 ± 4.3	5.7 ± 4.8	3.4 ± 4.5	2	H = 31.0	**0.001** ^**a**^
HAM-D, mean (SD)	18.8 ± 5.6	10.0 ± 6.4	3.0 ± 1.7	2	H = 37.1	**0.001** ^**b**^
SCID Depression positive (*n*)	14	7	NA	1	*X*^2^ = 11.0	**0.001**
UCLA-3, mean (SD)	56.11 ± 8.2	36.31 ± 4.90	31.30 ± 5.90	2	H = 42.2	**0.001** ^**b**^
Antidepressant use						
None, *n*	10	20	20	NA	NA	NA
SSRI monotherapy, *n*	5	4	0	NA	NA	NA
Combination antidepressant therapy, *n*	3	2	0	NA	NA	NA

*F, female; M, male; SD, standard deviation; ICG, Inventory of Complicated Grief; MMSE, Mini-Mental State Exam; DRS-2, Dementia Rating Scale-2; CIRS-G, Cumulative Illness Rating Scale-Geriatric; HAM-A, Hamilton Anxiety Rating Scale; HAM-D, 17-item Hamilton Depression Rating Scale; SCID, Structured Clinical Interview for DSM-5 Disorders; SSRI, selective serotonin reuptake inhibitor; SNRI, serotonin norepinephrine reuptake inhibitor; NA, not applicable; FET, Fisher's exact test*.

*For categorical data, statistical significance was evaluated using Chi-square test; For continuous variables, significance was evaluated using Kruskal-Wallis H-test (2 or 3 groups)*.

a,b *Post-hoc analyses revealed the source of Kruskal-Wallis (^a^Grief-High Loneliness vs. health comparison, and Grief-High Loneliness vs. Grief-Low Loneliness groups; ^b^Differences among all three groups). p < 0.05 is shown in bold*.

### Cross-Sectional Findings

**Figure 1:**
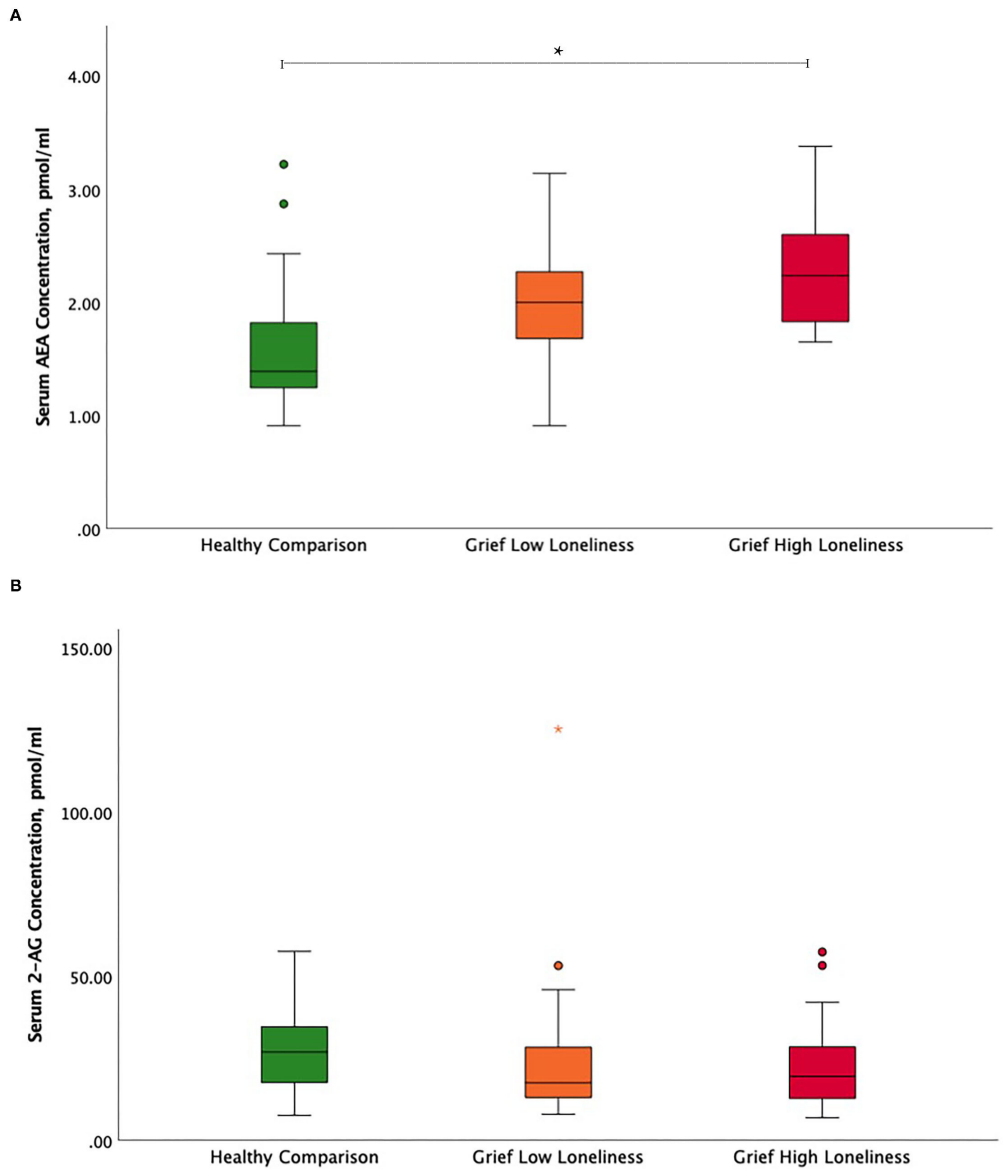
Circulating endocannabinoid concentration differences between high and low lonely grief and healthy comparison groups. Illustration of serum **(A)** AEA and **(B)** 2-AG group differences. In the boxplot, data indicated by circles and asterisks were outlier and extreme outliers, respectively. The asterisk depicts the significant finding. AEA, *N*-arachidonoylethanolamine or anandamide; 2-AG, 2-arachidonoylglycerol.

The ANCOVA showed significant serum AEA concentration differences between HC, Grief-LL, and Grief-HL groups, after adjusting for age and gender (*F* = 9.62, *p* < 0.001) Serum AEA concentrations were significantly increased in the Grief-HL group compared with HC (*p* = 0.001), but not in the Grief-HL group compared with the Grief-LL group (*p* = 0.72) (Figure 1A). Serum 2-AG (*F* = 0.27; *p* = 0.77; Figure 1B) and cortisol (*F* = 0.048; *p* = 0.95; Supplementary Figure 1) concentrations did not differ among the three groups.

In grief participants, linear regression analysis revealed a positive association between loneliness scores and serum AEA concentrations after adjusting for age, gender, and TSL (partial *r* = 0.36; *p* = 0.02). In model 2, these findings remained significant after adding ICG as a covariate (partial *r* = 0.33, *p* = 0.03) but were no longer significant after also adjusting for HAM-D (partial *r* = 0.28; *p* = 0.09) (Table 2). The relationship between loneliness scores and serum 2-AG concentrations was not significant (Supplementary Table 1). Exploratory analyses did not show interaction effects of loneliness and ICG, and loneliness and HAM-D, respectively, with AEA and 2-AG.

**Table 2:** Cross-sectional relationship between loneliness and circulating AEA concentrations in grieving older adults.

**Model**	**Partial *r***	**df**	***t*-value**	***p*-value**
Model 1	0.36	1	2.43	0.02
Model 2	0.33	1	2.16	0.03
Model 3	0.28	1	1.74	0.09

*Model 1, adjusted for age, gender, and TSL; Model 2, Adjusted for age, gender, TSL, and ICG; Model 3, Adjusted for age, gender, TSL, ICG, and HAM-D*.

*AEA, N-arachidonoylethanolamine or anandamide; df, degree of freedom; TSL, Time since loss; ICG, Inventory of Complicated Grief; HAM-D, 17-item Hamilton Depression Rating Scale. p < 0.05 is shown in bold*.

The correlation between cortisol and AEA concentrations was insignificant (partial *r* = −0.15; *p* = 0.23; data not shown) while the correlation between cortisol and 2-AG was positive and showed an insignificant trend (partial *r* = 0.199; *p* = 0.11) (Supplementary Figure 2).

### Longitudinal Findings

**Figure 2:**
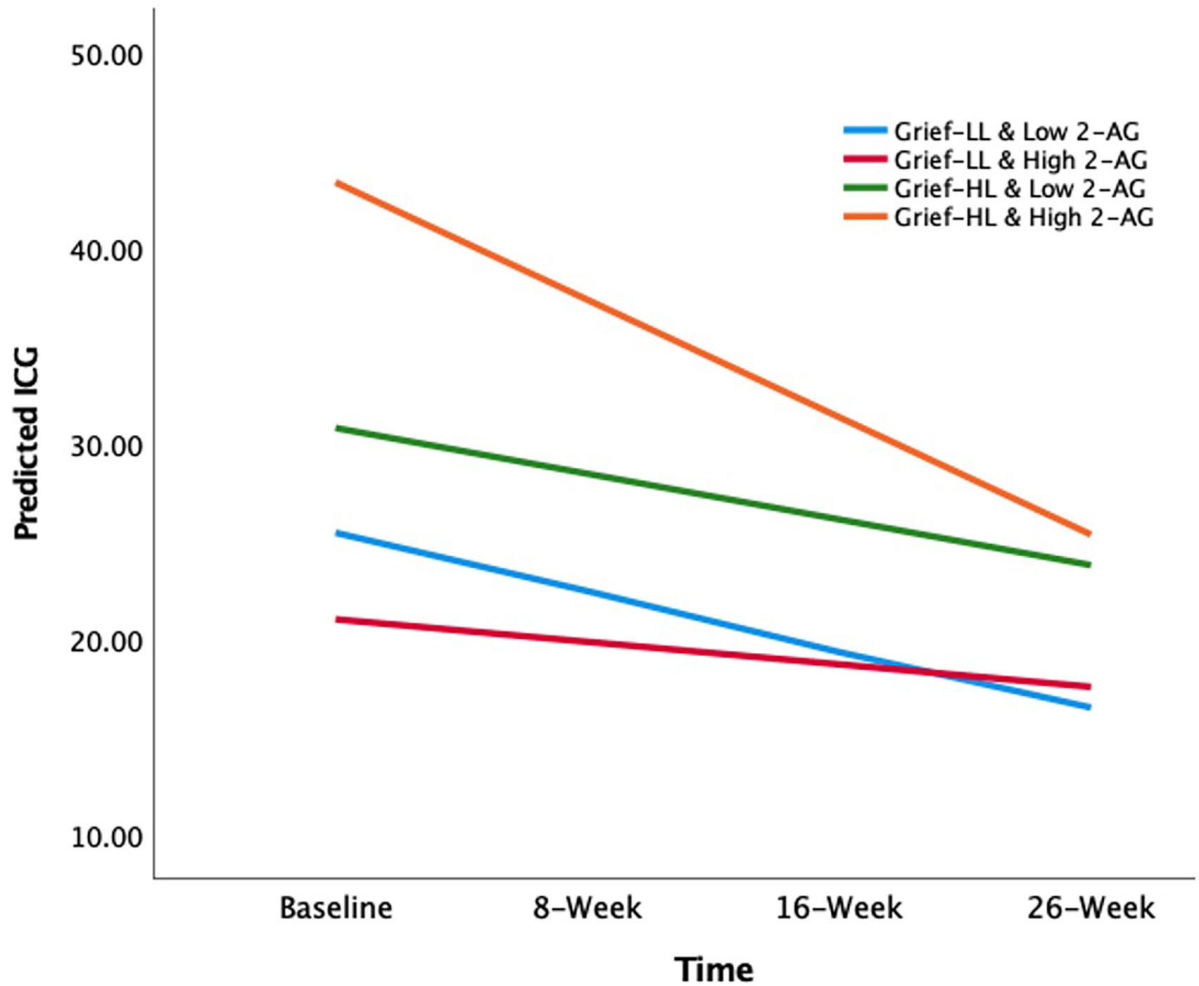
The effects of circulating 2-AG concentration on the association between baseline loneliness and longitudinal grief symptom trajectories. The X-axis depicts the time of longitudinal visits; Y-axis depicts the mean predicted ICG scores. The lines are the slopes for each group (color): −2.98 (low loneliness and low 2-AG group; light blue); −1.15 (low loneliness and high 2-AG group; red); −2.34 (high loneliness and low 2-AG group; green); −6.00 (high loneliness and high 2-AG group; orange). 2-AG, 2-arachidonoylglycerol; ICG, Inventory of Complicated Grief.

In grief participants, mixed-effects linear regression analysis showed that the loneliness-by-2-AG interaction was positively associated with ICG score at baseline (β estimate: 20.64; 95% CI: 0.95 to 40.33; *p* = 0.04). Also, ICG scores were found to decrease over 26 weeks (β estimate: −2.98; 95% CI: −5.14 to −0.81; *p* = 0.01). The 3-way loneliness-by-2-AG-by-visit interaction term was significant, i.e., the loneliness-by-2-AG interaction was associated with decreases in the ICG scores over 26 weeks (β estimate: −5.49; 95% CI: −10.31 to −0.66; *p* = 0.03) (Table 3). The slopes of ICG from baseline to 26 weeks were as follows: −2.98 (low loneliness/low 2-AG); −1.15 (low loneliness/high 2-AG); −2.34 (high loneliness/low 2-AG); and −6.00 (high loneliness/high 2-AG) (Figure 2). The grief participants with high loneliness at baseline and high serum 2-AG concentrations had a greater rate of improvement in ICG scores over 26 weeks compared with the other three groups; i.e., low loneliness/low 2-AG (*X*^2^ = 8.28, *p* = 0.004); low loneliness/high 2-AG (*X*^2^ = 13.25, *p* < 0.001); and high loneliness/low 2-AG (*X*^2^ = 11.01, *p* < 0.001), respectively (Figure 2). The mixed-effects linear model that examined the three-way interaction of baseline loneliness, baseline AEA, and visits with longitudinal ICG scores, controlling for age, gender, TSL, main, and covariate-by-visit interaction terms were not significant (β estimate: −1.44; 95% CI: −6.59 to 3.70; *p* = 0.58) (Supplementary Table 2).

**Table 3:** Linear mixed-effects model exploring the moderating effects of circulating 2-AG concentrations on the relationship between baseline loneliness and grief symptom (ICG) trajectories.

**Model**	**β Estimate (95% Cl)**	**df**	***t*-value**	***p*-value**
Age	0.10 (−0.30 to 0.49)	38	0.49	0.63
Gender	0.03 (−7.64 to 7.70)	38	0.01	0.99
Time Since loss (days)	0.01 (−0.03 to 0.05)	38	0.63	0.54
Loneliness (high/low)	5.96 (−7.90 to 19.82)	55	0.86	0.39
2-AG (high/low)	−6.00 (−18.26 to 6.26)	55	−0.98	0.33
Visit/time	−2.98 (−5.14 to −0.81)	114	−2.72	**0.01**
Loneliness-by-2-AG interaction	20.64 (0.95 to 40.33)	55	2.10	**0.04**
Loneliness-by-time interaction	0.64 (−2.74 to 4.03)	114	0.38	0.71
2-AG-by-time interaction	1.83 (−1.30 to 4.95)	114	1.16	0.25
Loneliness-by-2-AG-by-visit interaction	−5.49 (−10.31 to −0.66)	114	−2.25	**0.03**

*2-AG, 2-arachidonoylglycerol; ICG, Inventory of Complicated Grief. p < 0.05 is shown in bold*.

## Discussion

This study showed that grieving individuals reporting high loneliness have increased circulating AEA concentrations compared to their non-bereaved healthy counterparts, while neither cortisol nor 2-AG concentrations differed among the groups. Cross-sectionally, loneliness scores were positively associated with serum AEA concentrations in grievers; however, this significance was lost after accounting for depression severity. In grieving individuals, the association between high loneliness at baseline and faster amelioration of grief symptoms over 6 months (i.e., 26 weeks) was seen in those with high circulating 2-AG concentrations. Our preliminary data supports hypothesis generation that in grieving elders who endorse high loneliness early on after bereavement, increased circulating eCB concentrations–measures reflective of an efficient ECS system–hasten the resolution of grief symptoms and facilitate better adaptation to the loss.

Loneliness, one of the most intense and distressing symptoms reported after bereavement, is an important predictor of perceived psychological stress in later life (39). An efficient ECS system is stress-responsive, which in turn leads to less physical and emotional distress. Our cross-sectional findings thus lead us to hypothesize that increased circulating eCBs seen in lonely grieving individuals are reflective of an efficient and normal functioning “stress-responsive” ECS system. In support of this notion, we previously showed that grievers have elevated circulating AEA concentrations compared with their non-bereaved healthy counterparts (20). Increases in circulating AEA concentrations to acute psychological and physical stress exposure have also been previously demonstrated in humans (15, 40, 41). Moreover, multiple investigations have shown that moderate levels of sustained aerobic exercise increase circulating AEA concentrations in healthy individuals (16, 19).

The positive cross-sectional association between loneliness and AEA concentrations in bereaved elders appears to be driven by depression severity. Multiple studies have previously shown increases in circulating AEA concentrations in minor and major depression (42, 43), although this finding is not universal (44). The AEA finding discrepancies in depression could be related to the duration of depressive episodes. For instance, decreases in circulating eCBs were associated with chronicity of depressive symptoms in major depression in one study (42). Thus, a diminished ECS system activity may reduce buffering against stress and contribute to more chronic and persistent depressive symptoms. When one-third of acute grievers experience major depression 1 month after bereavement, only about 15% have chronic, persistent depression at 1 year (45). In our study, about one-half of the grief participants met bereavement-related major depression criteria. Since most grieving elders in our study were within a few months post-loss, it is possible that their depression would resolve and not become chronic. A more fine-grained statistical approach using data comprising longitudinal loneliness, circulating eCB, and depressive and grief symptom assessments from acutely grieving older adults is vital to better understand their interrelationships in future studies.

High levels of loneliness at baseline are associated with faster resolution of grief symptoms over 6 months, but only in those with increased 2-AG concentrations. Aerobic exercise-induced 2-AG increases are found to correlate with positive mood outcomes in young healthy individuals (17), and in patients with major depression (46). Our novel longitudinal findings, together with the data outlined above, thus suggest that an activated ECS system (reflected by increased circulating 2-AG mobilization) in response to bereavement may be crucial for better adaptation to attachment loss and to ease the transition to integrated grief in bereaved elders who report high loneliness. Extrapolating from preclinical (9, 24) and PTSD studies (18, 25) showing a blunted ECS system response following stress exposure, it is plausible that lower circulating eCB concentrations in intensely lonely grieving older adults are associated with persistent grief symptoms. Moreover, future investigations should examine if lower eCB concentrations, which may reflect a blunted ECS response, in lonely grieving older adults are associated with development of PGD, a trauma-related disorder that causes immense suffering and disability.

The brain mechanisms by which serum eCB concentrations associate with grief symptom trajectory in lonely, grieving elders are unknown. We suggest possible mechanistic theories. The ECS system affects emotion regulation and reward processing brain circuit function, and fear learning and extinction (8). Preclinical evidence demonstrates that CB1 receptors are widely expressed in emotion regulation brain regions (e.g., the amygdala, prefrontal cortex, etc.). Brain concentrations of the endogenous CB1 receptor ligands, AEA and 2-AG, are sensitive to stress; and enhanced CB1 receptor signaling during stress dampens the emotion regulation brain circuit function, resulting in an accelerated termination of behavioral stress responses (8). In contrast, chronic stress in rodents results in reduced eCB-mediated CB1 receptor signaling, and poor adaptation and excessive stress responses (47). In other words, reduced eCB signaling may lead to psychopathologies linked to emotion regulation, including PGD. Interestingly, we recently demonstrated that enhanced amygdala-frontal functional connectivity in bereaved elders was associated with worsening grief symptoms over 6-months (48). Stress can also potentiate ECS system activation of the reward processing brain circuit, which can be detrimental in some situations (49, 50). In PGD, it is hypothesized that the death of an attachment figure (i.e., a close family member) leads to disrupted signaling in the reward processing brain circuit and the onset and persistence of yearning, a core PGD symptom (51). In PGD, nucleus accumbens hyperactivity to deceased-related stimuli were found to associate with yearning (52). The relationship between circulating eCB concentrations and emotion regulation and reward processing brain circuit function in lonely, bereaved elders, and how their interrelationships contribute to PGD development remain to be elucidated.

HPA axis dysregulation is observed following bereavement. Higher cortisol concentrations are consistently reported during the early bereavement period than in non-bereaved adults (26, 53, 54). However, the cortisol findings in PGD are more variable (27, 55, 56), with one study reporting lower morning and overall diurnal cortisol levels (56). Loneliness is also associated with cortisol dysregulation, though the loneliness-HPA axis link following an acute stress like bereavement is unclear (57). We did not find serum cortisol differences among the groups. However, since cortisol assessment was not our primary goal, we did not control for time of day in our blood draws, and cortisol concentrations were quite variable. We did not find a significant association between cortisol and AEA concentrations, though the correlation between cortisol and 2-AG concentrations showed a trend toward a positive correlation. These findings are in accord with preclinical data demonstrating that cortisol elevates brain 2-AG concentrations (58) and recent data in humans (59). Since it is hypothesized that PGD is associated with dysfunctional stress responding, future investigations should examine the impact of HPA axis activity in the development of PGD, and the link between cortisol and circulating eCB concentrations in bereaved older adults.

## Limitations

Our study had a modest sample size and participants were predominantly white and female; our findings are for hypothesis-generating purposes and should be interpreted with caution. We explored associations between loneliness and grief symptoms; thus, causality cannot be inferred. We only collected loneliness and circulating eCBs at baseline; future investigations should examine how changes in loneliness and eCBs are related to grief trajectories. Also, the effect modifying role of objective social connection variables (e.g., living alone, social support, social network size, etc.) on the loneliness-grief symptom association might provide unique insights. Close to one-half of our grief participants met DSM-5 criteria for bereavement-related major depressive disorder. We, therefore, repeated our longitudinal analyses by extending the mixed-effects model by including baseline HAM-D scores as a covariate; our findings remained significant. Future adequately powered studies should account for depression changes over time while examining the moderating role of eCBs on the longitudinal loneliness-grief association. A recent study showed that circulating eCBs are associated with antidepressant therapy (43). We did not include antidepressant use as a covariate to limit the number of regressors in this study with modest sample size. Some grief participants may have been attending individual or group psychotherapy sessions; details regarding these non-pharmacologic interventions were not available. Time since bereavement and time of blood collection are other factors that can alter our findings and should be accounted for in data collection and/or statistical models. Finally, lifestyle factors, particularly physical exercise, can alter circulating eCB concentrations. Information regarding acute and chronic physical activity routines of participants were not collected as part of this study.

## Conclusion

This pilot, hypothesis-generating study demonstrates that circulating eCB concentrations are increased in grieving elders who endorse high loneliness. The association between high levels of loneliness at baseline and faster amelioration of grief symptoms over 6 months was moderated by high circulating 2-AG concentrations. Our results lead us to hypothesize that increased circulating eCB concentrations, which are reflective of an efficiently functioning ECS system, protect severely lonely grieving elders from PGD development.

## Data Availability Statement

The raw data supporting the conclusions of this article will be made available by the authors, without undue reservation.

## Ethics Statement

The studies involving human participants were reviewed and approved by Institutional Review Board at the Medical College of Wisconsin. The patients/participants provided their written informed consent to participate in this study.

## Author Contributions

MK, LB-M, CH, and JG: conception and design. SC, JG, and GS: acquisition of data. MK, LB-M, TM, SC, N-OB, GS, CR, CH, and JG: analysis and interpretation of data. MK, LB-M, TM, CH, and JG: drafting the original version. MK, LB-M, TM, SC, N-OB, GS, CR, CH, and JG: revising and editing the manuscript critically for important intellectual content, and gave final approval. All authors contributed to the article and approved the submitted version.

## Funding

This work was supported in part by the National Institutes of Health [R01 MH122490 and R21 MH109807 (JG)]; the Costigan Family Foundation (JG); the Kubly Fund for Depression Research (CH); Stephen T. Sexton Memorial Foundation; and the National Center for Advancing Translational Sciences, National Institutes of Health, Award Number UL1TR001436.

## Conflict of Interest

CH has equity and is on the Scientific Advisory Board for Formulate Biosciences and is on the Scientific Advisory Board for Phytecs, Inc. The remaining authors declare that the research was conducted in the absence of any commercial or financial relationships that could be construed as a potential conflict of interest.

## Publisher's Note

All claims expressed in this article are solely those of the authors and do not necessarily represent those of their affiliated organizations, or those of the publisher, the editors and the reviewers. Any product that may be evaluated in this article, or claim that may be made by its manufacturer, is not guaranteed or endorsed by the publisher.
