# Supplementary material for: Loneliness, Circulating Endocannabinoid Concentrations, and Grief Trajectories in Bereaved Older Adults: A Longitudinal Study
Source: Front Psychiatry. 2021 Dec 8;12:783187. doi: 10.3389/fpsyt.2021.783187 (PMC8692767; doi:10.3389/fpsyt.2021.783187)
Supplement: Supplementary file 1 [file Data_Sheet_1.docx]

**SUPPLEMENTARY MATERIAL**

**Table S1.** Cross-sectional relationship between loneliness and circulating 2-AG concentrations in bereaved older adults.

| **Model** | **Partial r** | **df** | ***t* Value** | ***P* Value** |
| --- | --- | --- | --- | --- |
| Model 1 | 0.24 | 1 | 1.54 | 0.13 |
| Model 2 | 0.21 | 1 | 1.33 | 0.19 |
| Model 3 | 0.12 | 1 | 0.70 | 0.49 |

**Footnote.** Partial correlations of loneliness with 2-AG. **Footnote.** Model 1: adjusted for age, gender and TSL; Model 2: Adjusted for age, gender, TSL and ICG; Model 3: Adjusted for age, gender, TSL, ICG and HAM-D. Excludes one outlier with 2-AG concentrations =125 pmol/ml**.** Abbreviations. 2-AG: 2-arachidonoylglycerol; df: degree of freedom; TSL: Time since loss; ICG: Inventory of Complicated Grief; HAM-D: 17-item Hamilton Depression Rating Scale.

**Table S2.** **Linear mixed-effects model exploring the moderating effects of circulating AEA concentrations on the relationship between baseline loneliness and grief symptom (ICG) trajectories.**

| **Model** | **Β Estimate (95% Cl)** | **df** | **t** | **P-value** |
| --- | --- | --- | --- | --- |
| Age | 0.10 (-0.29 to 0.48) | 38 | 0.51 | 0.62 |
| Gender | 0.14 (-7.56 to 7.84) | 38 | 0.04 | 0.97 |
| Time Since Loss (Days) | 0.02 (-0.02 to 0.06) | 38 | 1.11 | 0.27 |
| Loneliness (High/Low) | 6.68 (-8.94 to 22.30) | 60 | 0.86 | 0.40 |
| AEA (High/Low) | -4.54 (-16.60 to 7.52) | 59 | -0.75 | 0.45 |
| Visit/Time | -2.33 (-4.51 to -0.16) | 117 | -2.13 | 0.04 |
| Loneliness-by-AEA Interaction | 15.41 (-3.87 to 34.68) | 59 | 1.60 | 0.11 |
| Loneliness-by-Visit Interaction | -1.13 (-5.06 to 2.80) | 117 | -0.57 | 0.57 |
| AEA-by-Visit Interaction | 0.44 (-2.79 to 3.66) | 117 | 0.27 | 0.79 |
| Loneliness-by-AEA-by-Visit Interaction | -1.44 (-6.59 to 3.70) | 117 | -0.55 | 0.58 |

Abbreviations. AEA: N-arachidonoylethanolamine or anandamide; ICG: Inventory of Complicated Grief

**Supplementary Figure S1. Serum Cortisol concentration differences between high and low lonely**

**grief and healthy comparison groups.** In the boxplot, data indicated by a circle was an outlier.


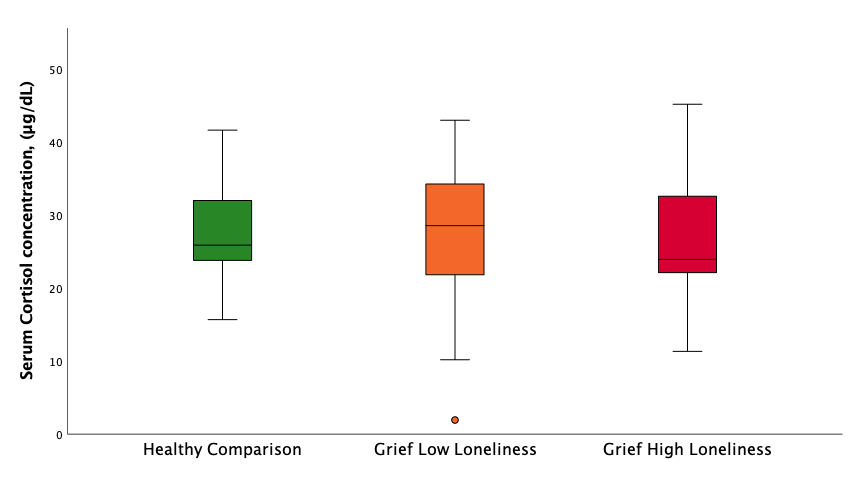


**Supplementary Figure S2. Association between serum cortisol concentrations with (A) AEA and (B) 2-AG concentrations without covariate adjustment.** Abbreviation: AEA: N-arachidonoylethanolamine or anandamide; 2-AG: 2-arachidonoylglycerol.


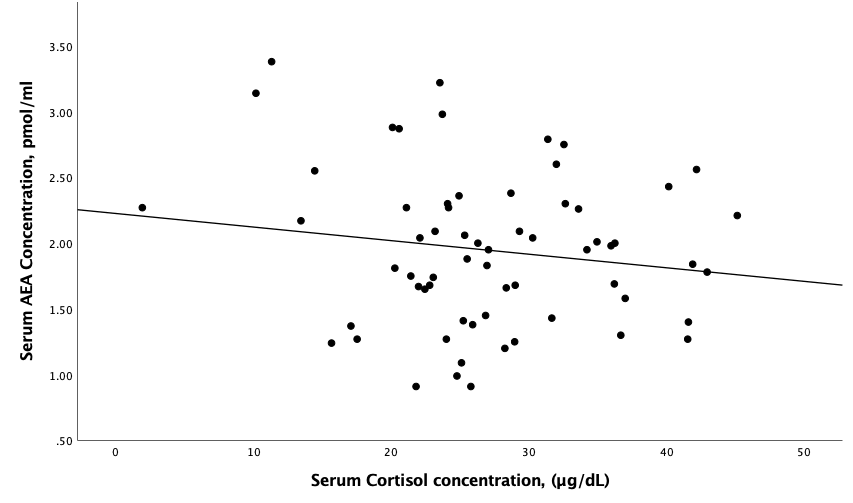

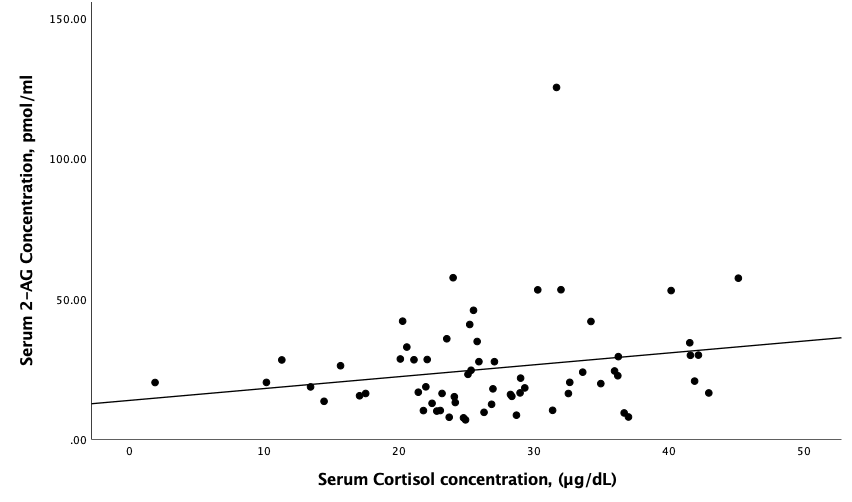


**A**

**B**
